# Utilizing Naloxone Education to Reduce the Mortality and Morbidity Rate of Overdose Deaths Within Opioid-Exposed Populations

**DOI:** 10.7759/cureus.84793

**Published:** 2025-05-25

**Authors:** Anna M Marchek, Julia P Wolf, Georgia H O'Leary, Gabriela Pages, Madison C Benefield, Brooke Bennett, Priyanka Arunkumar, Marc Burrows, David Redden, Alexis Stoner, Jeff Cashman

**Affiliations:** 1 Medical School, Edward Via College of Osteopathic Medicine, Spartanburg, USA; 2 Public Health, Challenges, Inc., Powdersville, USA; 3 Research and Biostatistics, Edward Via College of Osteopathic Medicine, Auburn, USA; 4 Epidemiology and Public Health, Edward Via College of Osteopathic Medicine, Spartanburg, USA; 5 Family Medicine, Edward Via College of Osteopathic Medicine, Spartanburg, USA

**Keywords:** naloxone, naloxone education, narcan, opioid overdose, overdose prevention

## Abstract

Objectives: While efforts are being made to reduce opioid overdose fatalities, there is a need to equip individuals to act in overdose emergencies in an effort to slow the increasing rates of preventable opioid-related deaths in the United States. This study sought to determine whether education on naloxone administration would increase confidence in individuals utilizing harm reduction services to intervene in the presence of an opioid overdose.

Methods: A cross-sectional study was conducted that included adult participants attending Challenges, Inc., a mobile harm reduction service site. Participants completed a baseline survey assessing their level of comfort and experience with naloxone administration. An optional standardized educational session focusing on proper naloxone use followed, and all participants were offered free naloxone. Participants completed a post-survey reassessing their confidence and willingness to administer naloxone.

Results: Of the 100 participants, 75% reported witnessing at least one opioid overdose, with the majority of those seeing 10 or more opioid overdoses. Additionally, 58% of respondents had previously administered naloxone treatment, on average between one and four times. A significant increase was found in participants' confidence level of administering naloxone following the educational session (p < 0.0001). When asked about the likelihood of intervening when witnessing an opioid overdose, 96% agreed or strongly agreed that they would administer naloxone treatment.

Conclusion: A single education session increased the confidence, willingness, and comfort of individuals in administering naloxone in the presence of an opioid overdose. Ultimately, improving naloxone education and access could lead to a decrease in morbidity and mortality in association with opioid-related overdoses.

## Introduction

Opioid-involved overdose deaths in the United States have dramatically increased from 49,860 in 2019 to 81,806 in 2022 [1]. The opioid crisis is an evolving issue, and more education initiatives and harm reduction strategies are needed [2]. South Carolina specifically has seen an alarming 25% increase in overdose deaths from 2020 to 2021, with opioids as the primary cause [3]. A recent review found that education and naloxone distribution improve both long-term knowledge regarding opioid overdoses and participants' attitudes toward naloxone, as well as provide adequate training to manage overdoses [4]. 

Naloxone is an opioid-receptor antagonist that can reverse overdoses from opioids, including heroin, fentanyl, and prescription opioids, when administered in a timely manner [5]. Historically, this drug was used in emergency medical settings in opioid overdose cases [6]. It is now readily available over the counter in the United States and can be given through either the intranasal or intramuscular route. This is particularly important in the setting of an overdose where medical access may not be available and intervention is needed by a bystander [7]. 

Unfortunately, naloxone is not fully being utilized due to current barriers to access, stigma surrounding its possession and usage, and improper education on administration [6]. A study analyzing the out-of-pocket costs for naloxone demonstrated a 606% increase in out-of-pocket cost of naloxone for uninsured patients between 2014 and 2018 [8]. In general, the cost of naloxone without a prescription averages around $62.67 [9]. Not only is this a major expense, especially for those who are uninsured, but its availability in pharmacies varies and is not guaranteed. In addition, stigma surrounding naloxone usage is associated with the belief that administering naloxone encourages continued opioid use and deters individuals from seeking treatment [10,11]. Furthermore, many people are afraid to obtain naloxone over the counter due to its association with opioids [12-14]. Finally, there is hesitancy for intervention among bystanders who either have not been educated on its use or fear retaliation from inconsistent state laws [15]. Therefore, there is an urgent need to increase accessibility and provide proper education on the administration of this antidote to reverse opioid overdoses [16,17].

The objective of this study was to investigate whether targeted education on naloxone administration instills confidence and readiness to act during opioid overdose scenarios among individuals utilizing harm reduction services. This primary objective is grounded in the urgent public health need to reduce opioid-related deaths through timely bystander intervention and effective naloxone use. In addition, this study sought to assess two secondary objectives, including assessing the presence and influence of the bystander effect and evaluating participants' prior experience with naloxone and exposure to overdose situations. The bystander effect is a phenomenon in which an individual's likelihood of helping decreases when passive bystanders are present in a critical situation. This is especially important, as bystander administration of naloxone is critical in mitigating opioid overdose mortality [18]. The rate of overdose exposure and prior naloxone experience was also studied to gain an understanding of the level of risk involved within a more representative sample of individuals actively seeking harm reduction services. These factors are critical to understanding barriers to intervention and tailoring future educational initiatives.

This study was conducted in partnership with Challenges, Inc., a mobile overdose prevention and harm reduction service based out of Greenville, South Carolina. Previous pilot research done by our team explored this topic within treatment-seeking and healthcare populations. However, this sample population, along with many of the other studies that have been conducted, has not fully accounted for the high-risk populations that would benefit most from these services. Therefore, our research uniquely focuses on individuals actively engaged with harm reduction services who would benefit most from naloxone education. By capturing insights from this representative population, our findings have the potential to inform more inclusive and impactful public health strategies.

## Materials and methods

This study utilized a cross-sectional pre- and post-study design and was approved as exempt by the Edward Via College of Osteopathic Medicine Institutional Review Board. 

Participants included adults seeking services at a weekly Challenges, Inc. mobile unit site from January to May 2024. Challenges, Inc. offers free harm reduction resources including HIV and hepatitis C rapid testing, clean syringes, fentanyl and xylazine test strips, cotton filters, cookers, alcohol swabs, tourniquets, sharps containers, Department of Public Health (DPH) biohazard labels [19], and intramuscular and intranasal naloxone [20]. 

Consented participants were invited to complete an intake questionnaire and were categorized into two groups based on their preference to receive naloxone education: those who agreed to training and those who declined. The intake survey collected demographic information (age, sex, gender), accessibility of the service location, and prior experience with opioid overdoses, naloxone administration, and naloxone education. Survey questions related to opioid exposure were developed with a board-certified family medicine physician with expertise in addiction medicine and harm reduction. Participants also rated their baseline confidence in administering naloxone using a 5-point Likert scale, where 1 indicated low confidence and 5 indicated high confidence [21-23]. To reduce interviewer bias, all participants were given the opportunity to complete the survey privately and independently. In addition, to further minimize social desirability bias and protect participant privacy, all responses were collected anonymously and stored securely without any identifying information.

Following the intake questionnaire, participants who opted for naloxone education received training on the proper administration of naloxone using standardized evidence-based content by medical student volunteers [24]. Each educational session lasted approximately five to 10 minutes and was personalized to each participant. Training sessions included recognizing signs of an opioid overdose, correct use of intranasal and intramuscular naloxone, importance of contacting emergency services, and guidance on redosing and post-administration care. Hands-on practice was facilitated using naloxone tester models.

After the educational session, participants in the education group completed the post-survey, while those in the non-education group proceeded directly to the post-survey. The survey included the same intake question measuring individual confidence in administering naloxone using a Likert scale. To assess the bystander effect, all participants answered a question regarding their willingness to administer naloxone in the event of an opioid overdose. This was also rated on a Likert scale indicating their agreement to the statement "If I saw an individual overdosing, I would administer naloxone/Narcan", with 1 being strongly disagree up to 5 being strongly agree. At the end of the survey, all participants were offered free intramuscular and/or intranasal naloxone, depending on the availability of supplies.

The primary outcomes assessed included changes in confidence administering naloxone and willingness to intervene (the bystander effect) in overdose situations following education. Changes in confidence administering naloxone were compared between the two groups, which included those who chose to be educated on proper naloxone use and those who were not. Another primary outcome measure assessed previous experience with naloxone and opioid overdose exposure to understand the level of risk within the study population. Secondary outcomes included the assessment of demographic and experiential factors that might influence the effectiveness of the educational intervention. Data were analyzed using SAS software, version 9.4 (SAS Inc., Cary, NC, USA) [25]. Continuous and semicontinuous variables were summarized using means and standard deviations, while categorical variables were analyzed using proportions and 95% confidence intervals appropriate for multinomial outcomes. The sign test, a nonparametric method, was employed to determine the significance of changes in participants' comfort levels with naloxone administration before and after the intervention. Statistical significance was determined at an alpha level of 0.05.

## Results

A total of 100 individuals utilizing the Challenges, Inc. resources were enrolled into the study. Of those enrolled, 72 individuals agreed to receive proper naloxone training, while 28 individuals chose not to. Baseline demographics including age ranges, sex, and race of participants are shown in Table 1. The majority of the individuals enrolled in the study were male (60%) and between the ages of 18 and 44 (70%), where 75% of the total participants identified as Caucasian and 16% identified as African American.

**Table 1:** Participant demographics Baseline demographics of participants including age, sex, and race. Values represent the number of respondents, with percentages shown in parentheses.

Demographics	n (%)
Age range (in years)	
18-24	6 (6%)
25-34	29 (29%)
35-44	35 (35%)
45-54	21 (21%)
55+	9 (9%)
Sex	
Male	60 (60%)
Female	40 (40%)
Race	
White	75 (75%)
Black or African American	16 (16%)
Other	9 (9%)

A majority of the participants (75%) indicated they had witnessed at least one opioid overdose, while only 57% of participants indicated prior experience administering naloxone (95% CI: 47.3-66.7%) (Table 2). Only 54% of participants received prior education on proper naloxone administration, with the highest percentage seen in participants aged 25-34 (71%) and 45-54 (67%) and the lowest in those aged 55+ (11%) (Table 3). Previous experience with naloxone administration was significantly associated with the age of the respondent (p < 0.001) and gender (p = 0.02), but not with race (p = 0.06). Notably, a higher percentage of female participants (62%) received prior education compared to their male counterparts (49%). These associations suggest that age and gender may influence naloxone exposure or use, potentially acting as variables in evaluating the effect of education. Age, gender, and race were not significantly associated with the frequency of delivering naloxone treatment. While age and gender were not significantly associated with the number of opioid overdoses witnessed, Caucasian respondents (75%) were significantly more likely to report witnessing an overdose compared to African American respondents (16%) (p = 0.02).

**Table 2:** Bystander response measure Frequency distribution of bystander measures reported by participants (n = 100), including the number of witnessed opioid overdoses and instances of naloxone administration. Values represent the number of respondents, with percentages shown in parentheses.

Amount (frequency)	0	1-4	5-9	10+
Witnessed opioid overdose	25 (25%)	28 (28%)	16 (16%)	31 (31%)
Administered naloxone	42 (42%)	31 (31%)	13 (13%)	14 (14%)

**Table 3:** Naloxone education exposure by age group and gender Responses to naloxone exposure survey questions, including prior naloxone education (n = 98) and agreement to receive naloxone education (n = 100), stratified by age group and gender. Totals are listed at the bottom of the table. Two respondents (one male aged 25-34 and one female aged 35-44) did not answer the prior education question but indicated experience administering naloxone. Values represent the number of respondents, with percentages shown in parentheses as n (%).

Prior naloxone education	Yes	No
Total	53 (54%)	45 (48%)
Age range (in years)		
18-24	3 (50%)	3 (50%)
25-34	20 (71%)	8 (29%)
35-44	15 (44%)	19 (56%)
45-54	14 (67%)	7 (33%)
55+	1 (11%)	8 (89%)
Gender		
Male	29 (49%)	30 (51%)
Female	24 (62%)	15 (38%)
Agreed to education	Yes	No
Total	72 (72%)	28 (28%)
Age range (in years)		
18-24	4 (67%)	2 (33%)
25-34	23 (77%)	6 (20%)
35-44	24 (69%)	11 (31%)
45-54	13 (62%)	8 (38%)
55+	8 (89%)	1 (11%)
Gender		
Male	45 (75%)	15 (25%)
Female	27 (68%)	13 (32%)

**Figure 1:**
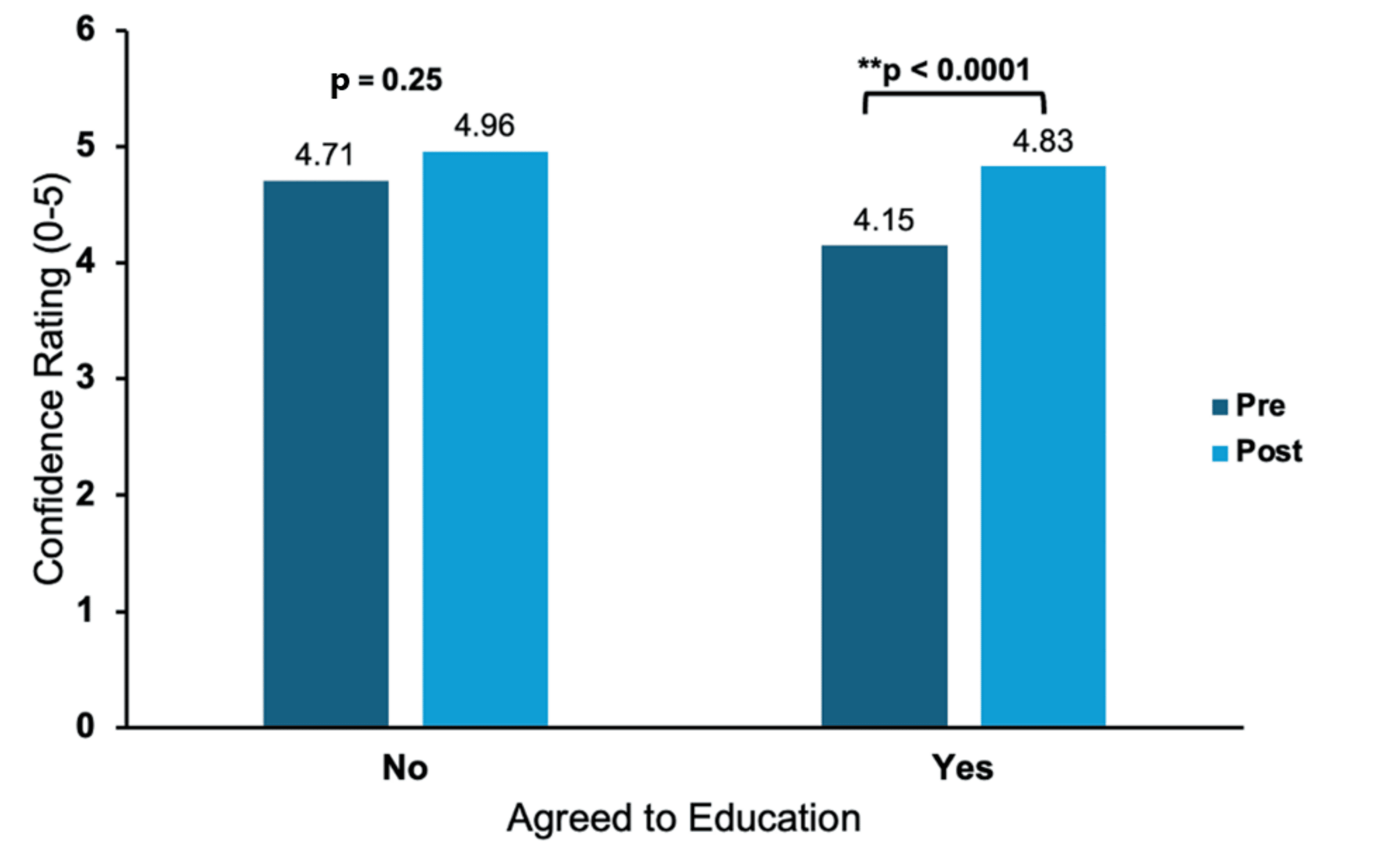
Ordinal measure of comfort administering naloxone comparing pre- vs. post-educational confidence Overall, there was a significant increase in confidence for naloxone administration following education from 4.15 (±1.08) to 4.83 (±0.41) (p < 0.0001). Those who did not choose to be educated did not have a significant change in confidence (p = 0.25). The majority of the patients who chose not to be educated had previous experience administering naloxone. **: significant p-value

Comfort rating for both groups on naloxone administration is shown in Figure 1. Results in the education group showed a significant increase in confidence following education on proper techniques of naloxone administration from 4.15 (±1.08) to 4.83 (±0.41) (p < 0.0001). Those who did not agree to be educated on proper naloxone administration did not have a significant change in confidence, starting with a baseline rating of 4.71 (±0.81) to 4.96 (±0.18) (p = 0.25). Of the 28 individuals who did not receive education, 27 had prior naloxone training, while one participant was not interested.

When measuring the bystander effect out of the 100 individuals enrolled, 96% agreed or strongly agreed with administering naloxone, 2% were neutral, and 2% disagreed or strongly disagreed with administering naloxone.

## Discussion

The purpose of this study was to determine whether personalized education on naloxone administration would increase bystander confidence and willingness to intervene in emergency situations, ultimately reducing opioid overdose mortality. This study focused on a high-risk opioid-prevalent population utilizing harm reduction services at the Challenges, Inc. mobile unit. Our study sought to target this population because a majority of the previous studies on naloxone education focused mainly on healthy controls rather than an opioid-involved population. In our study population, a majority of the participants had witnessed 10 or more opioid overdoses (31%); however, most participants overall (42%) had never administered naloxone (Table 2). This reveals a discrepancy between the frequency of witnessed opioid overdoses and the frequency of naloxone utilization, which may be explained by a lack of naloxone education, discomfort administering naloxone, or limited access to naloxone. Therefore, by increasing access, education, and confidence using naloxone, we can empower individuals to intervene with naloxone utilization and prevent opioid-related overdoses. Although this study population consists of individuals accessing harm reduction services, potentially limiting generalizability to the broader public, it remains highly relevant, as people who use opioids are disproportionately affected by overdose and have been shown to administer naloxone at significantly higher rates than other groups [26]. As such, studying this population provides critical insight into real-world naloxone use and informs broader public health strategies in the context of the US overdose crisis.

We also found a statistically significant increase in confidence levels among the participants who received proper naloxone training. It is clear that harm reduction strategies, such as naloxone administration education, have reduced mortality associated with opioid use. In particular, a study found that personally tailored opioid overdose prevention education and naloxone distribution increased knowledge of medication use for opioid use disorder and decreased perceived treatment barriers, further supporting our findings [27]. In addition, this study also reported an increased desire to quit all substances and a decrease in self-reported opioid use and overdose risk behaviors at follow-up [27]. Overall, these findings align with our results and support our hypothesis that education increases participants' confidence in administering naloxone in overdose situations. Additionally, the subgroup analysis revealed that the older age group (55+) was least likely to have received prior training, underscoring a potential demographic gap in naloxone preparedness. It should also be noted that a majority of individuals who declined naloxone education had previously received training. This may explain the lack of significant changes in pre-to-post confidence values and overall higher confidence ratings within the group that was not educated.

Interestingly, our results revealed that age and gender were significantly associated with prior naloxone education, while race approached significance. Specifically, we found that younger individuals (particularly those aged 25-34) and women were more likely to have received prior training (Table 3). These demographic factors may influence baseline exposure to naloxone education and should be considered when evaluating the impact of educational interventions. Additionally, while age and gender were not significantly associated with the number of opioid overdoses witnessed, Caucasian participants reported witnessing significantly more overdoses than African American participants. These trends may reflect differences in exposure, access to harm reduction resources, or structural determinants of health. Such variables likely influence participants' baseline familiarity with naloxone, and future studies should incorporate stratified analyses or multivariable models to better isolate the true effect of education on naloxone confidence and utilization.

Our study concluded that the majority of participants would be willing to intervene and administer naloxone if confronted with an opioid overdose. Nearly all participants agreed or strongly agreed that they would administer naloxone in an opioid-related emergency. This aligns with previous studies showing that bystander administration of naloxone is effective in treating overdose and proper training improves participants' knowledge of overdose recognition and management [28,29]. The small number of participants who were neutral or unwilling to administer naloxone could be explained by the limited supply of the preferred intranasal naloxone on the mobile harm reduction unit. This preference is consistent with other studies that found that 44% of participants chose the higher-dose nasal spray, 30% chose the intramuscular injection, and 26% chose the lower-dose nasal spray [18]. These findings emphasize the importance of expanding naloxone access and providing proper training to empower individuals to respond effectively in overdose emergencies.

Several limitations should be considered when interpreting the results of this study, including recall bias, social desirability bias, selection bias, and contextual limitations. First, recall bias may have affected participant responses, particularly regarding the number of opioid overdoses witnessed or past naloxone use. This may be due to the time elapsed since the event or cognitive effects related to substance use [30]. Future research should consider offering categorical response ranges rather than requiring specific numeric inputs to reduce this source of error. Second, social desirability bias may have influenced participants to overreport socially acceptable behaviors, such as their willingness to intervene during an overdose, or underreport behaviors perceived as negative, such as prior substance use [31]. This effect may have been amplified by the presence of researchers or staff during the survey; however, individuals were given the opportunity to complete the questionnaire independently and in private, which likely reduced this source of bias. Future studies could further address this by incorporating validated social desirability scales and consistently providing a private area for participants to complete questionnaires anonymously [32]. Third, selection bias may have occurred due to the self-selection of participants into the educational intervention group. Individuals who opted in may have had more interest in or prior exposure to naloxone use, potentially inflating the intervention's observed effect. This may limit the generalizability of the findings, especially to less-engaged populations; however, a majority of the participants enrolled desired naloxone education. Fourth, the study's context, a mobile harm reduction unit serving a specific geographic region, may reduce external validity, as results may not fully translate to other settings or populations with different access to resources or support services. Despite these limitations, the study offers valuable insights into the feasibility and immediate impact of tailored naloxone education in high-risk, real-world settings.

Future efforts should also be made to further investigate the association between age, gender, race, and their effect on opioid overdose prevention. This approach increases the education and access to naloxone throughout the population as a whole while also allowing for stratified comparisons. Finally, our study only assessed the bystander effect following naloxone education. Future studies should also include a survey question on the bystander effect prior to education to determine its significance in relation to naloxone training. 

## Conclusions

Through increasing accessibility to naloxone and providing naloxone education, we hope to empower individuals to intervene to decrease the overall morbidity and mortality associated with opioid overdoses. Providing these harm reduction services to high-risk opioid-using populations is essential to help reduce overdose fatalities. Overall, this study demonstrated that naloxone training instills confidence and willingness to act in individuals when faced with an opioid overdose. 
